# Risk Factors of All-Causes Mortality and *De Novo* Solid Malignancy Following Liver Transplantation: A Single-Center Survival Analysis in Iran

**DOI:** 10.34172/aim.34604

**Published:** 2025-08-01

**Authors:** Gholam Reza Sivandzadeh, Sara Shojaei-Zarghani, Seyed Ali Malek-Hosseini, Fardad Ejtehadi, Ramin Niknam, Omid Tarighat, Ali Reza Safarpour

**Affiliations:** ^1^Gastroenterohepatology Research Center, Shiraz University of Medical Sciences, Shiraz, Iran; ^2^Colorectal Research Center, Shiraz University of Medical Sciences, Shiraz, Iran; ^3^Abu Ali Sina Hospital for Medicine & Organ Transplant, Shiraz University of Medical Sciences, Shiraz, Iran

**Keywords:** Liver transplantation, Malignancy, Mortality, Survival

## Abstract

**Background:**

*De novo* solid tumors are considered major causes of mortality in liver transplant recipients. This retrospective cohort study aimed to assess the risk factors of all-causes mortality and *de novo* solid malignancy, as co-primary outcomes, following liver transplantation.

**Methods:**

The medical records of 2,600 patients who underwent liver transplantation at Abu-Ali Sina Charity Hospital in Shiraz, Iran, between 2010 and 2023, were evaluated to collect data of eligible patients. Cox proportional hazards regression was used to determine factors affecting mortality and *de novo* malignancy.

**Results:**

A total of 419 patients were included. Among them, 127 individuals (30.3%) died and 53 patients (12.6%) received a *de novo* solid malignancy diagnosis during the study period. The 1-, 5-, 10-, 15-, and 20-year survival rates of patients were 85%, 76%, 69%, 61%, and 58%, respectively, and the 1-, 5-, 10-, 15-, and 20-year proportion of patients free from *de novo* malignancy were 97%, 90%, 83%, 78%, and 78%. Age (hazard ratio [HR]=1.03, 95% confidence interval [CI]: 1.02 to 1.05, *P *value<0.001) and sirolimus (HR=0.44, 95% CI: 0.31-0.63, *P* value<0.001) were significantly associated with survival, and age (HR=1.05, 95% CI: 1.02-1.07, *P* value<0.001) and azathioprine (HR=5.85, 95% CI: 2.47-13.87, *P *value<0.001) were linked to an increased risk of *de novo* solid malignancy.

**Conclusion:**

Recipient’s age and immunosuppressive regimen are independently associated with mortality and malignancy development following liver transplantation. However, this study is limited by its retrospective design and single-center setting.

## Introduction

 Liver transplantation has been approved by the National Institutes of Health as a life-saving therapy for end-stage liver disease since 1983.^1^ It is estimated that 34,694 liver transplants have been performed globally in 2021, representing a 20% increase from 2015.^2^ Furthermore, between 1993 and 2016, 4,485 liver transplantations were performed in six Iranian centers, increasing steadily over this period, with Shiraz emerging as the leading national center.^3^ The short-term survival rate after liver transplantation has improved in recent years due to advancements in recipient and donor selection, post-operative care, and complication management. However, the long-term survival rate has not shown the same level of improvement.^4^ Infections, surgical complications, and cardiovascular diseases are the main causes of mortality in the early post-transplant period. However, malignancies (recurrence or *de novo*) are considered major causes of long-term mortality in liver transplant recipients.^5^

 It has been reported that patients who undergo solid organ transplantation face a 20-fold increased risk of developing *de novo* carcinoma compared to the general population.^6^ This elevated risk has been also observed in individuals following liver transplantation.^7^ Post-transplant lymphoproliferative disorders, as well as cancers affecting the head and neck, esophagus, colon, lung, cervix, and Kaposi’s sarcoma are among the prevalent* de novo* solid organ malignancies following liver transplantation.^7,8^ Several studies have identified post-transplant immunosuppressive treatments (especially calcineurin inhibitors and azathioprine), oncogenic viral infections, alcohol and tobacco use, and obesity and metabolic syndrome as potential risk factors contributing to this phenomenon.^7^ However, data on this topic are limited and controversial as some studies find no association between the type of immunosuppressive agents used and post-liver transplantation *de novo* malignancy.^9,10^ Therefore, the present study was conducted to assess all-causes mortality rates and the type and frequency of *de novo* solid organ malignancies occurrence following liver transplantation as well as their related risk factors among adult patients who underwent liver transplantation in a single center in Shiraz, Iran. The study was conducted at Abu-Ali Sina Charity Hospital in Shiraz, Iran, which performed 2,600 liver transplantations between 2010 and 2023, averaging approximately 200 transplants per year. This high volume establishes the center as one of the largest liver transplant centers globally,^11,12^ supporting its representativeness for this study.

## Materials and Methods

 This retrospective cohort study was conducted on all eligible patients who underwent liver transplantation in Abu-Ali Sina Charity Hospital in Shiraz, Iran, between March 21, 2010, and January 20, 2023, and whose medical records were available. The study utilized anonymized medical records and data, with no identifiable personal information reported; therefore, informed consent was not obtained. This manuscript was prepared in accordance with the STROBE (Strengthening the Reporting of Observational Studies in Epidemiology) guidelines.

###  Eligibility Criteria

 The inclusion criteria encompassed adult patients ( > 18 years old) who had been monitored for a minimum of six months after liver transplantation, underwent liver function tests at least annually post-transplantation, received transplant-related medications for at least six months, and had a minimum of six months elapsed between liver transplantation and cancer diagnosis. Patients were excluded if they had pre-transplant malignancy, died within the initial six months after liver transplantation, were diagnosed with cancer within the first six months after transplantation, or were identified with a recurrence or metastasis of prior malignancies.

###  Data Collection 

 The medical records of 2,600 liver transplant recipients were evaluated by a physician to sample eligible patients. Data pertaining to demographic (sex and age) and anthropometric characteristics, smoking and alcohol consumption, medical history, medication use, *de novo* malignancy diagnoses, mortality events, and corresponding dates were collected accordingly. Medication use was classified as binary (ever used/never used post-transplant). Adherence to medications was inferred from prescription records and follow-up notes, rather than being objectively assessed through patient evaluations. The causes of liver disease were classified according to clinical diagnoses. Comorbidities were defined based on the pre-transplant medical history and diagnoses. Malignancy diagnoses were based on pathology reports and clinical records.

###  Statistical Analysis

 The statistical analyses were conducted using the IBM SPSS software (version 26.0). Normality of the data was assessed using Kolmogorov-Smirnov test. Non-parametric quantitative and qualitative variables are presented as median (interquartile range [IQR]) and number (percentages), respectively. Mann-Whitney U test, chi-square, or Fisher’s exact tests were employed for between-group comparisons. Life table and Kaplan-Meier curve were utilized to evaluate overall survival rate and proportion of *de novo* malignancy, while Log-Rank test was used to detect significant differences between subgroups. Backward Cox proportional hazards regression was employed to determine factors affecting survival and *de novo *malignancy. The proportional hazards assumption was tested using Schoenfeld residuals, with no significant violations detected. Covariates were selected based on the literature and univariate significance (Log-rank test, *P* value < 0.10). Adjusted hazard ratio (HR) with a 95% confidence interval (CI) was reported, and a *P* value < 0.05 was considered significant.

## Results

**Figure 1:**
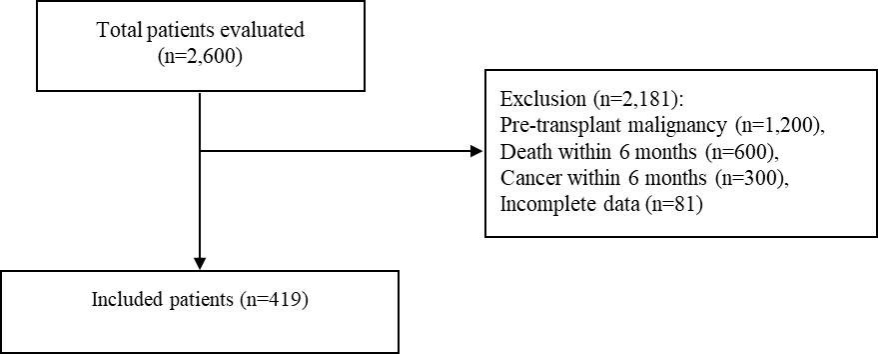
Study Selection Process

 Of the 2,600 liver transplant recipients evaluated, 2,181 were excluded based on predefined criteria (Figure 1). Finally, a total of 419 patients (51.8% male) with a mean age of 39.95 years were included. As shown in Table 1, the majority of these patients received a whole organ graft (94.3%). The major causes of liver disease among the included patients were hepatitis B virus (19.6%), autoimmune hepatitis (16.5%), cryptogenic (14.6%), and primary sclerosing cholangitis (12.9%). Prednisolone (100%), mycophenolate mofetil (97.9%), and tacrolimus (87.4%) were the most commonly used immunosuppressive drugs following transplantation.

**Table 1:** Patients’ Demographic and Clinical Characteristics

**Variable**	**Total (n=419)**	**Dead (n=127)**	**Alive (n=292)**	***P*** value^*^	**With ** ***de novo*** ** malignancy (n=53)**	**Without ** ***de novo*** ** malignancy (n=366)**	***P*** ** value**^†^
Sex, n (%)							
Male	217 (51.8)	70 (55.1)	147 (50.3)	0.369^a^	33 (62.3)	184 (50.3)	0.103^a^
Female	202 (48.2)	57 (44.9)	145 (49.7)		20 (37.3)	182 (49.7)	
Graft type, n (%)							
Whole organ	395 (94.3)	120 (94.5)	275 (94.2)	0.900^a^	51 (96.2)	344 (94.0)	0.754^b^
Split	24 (5.7)	7 (5.5)	17 (5.8)		2 (3.8)	22 (6.0)	
Cause of liver disease, n (%)^‡^				0.715^a^			**0.015**^a^
HBV	82 (19.6)	27 (24.1)	55 (20.2)		15 (29.4)	67 (20.1)	
Autoimmune hepatitis	69 (16.5)	18 (16.1)	51 (18.8)		5 (9.8)	64 (19.2)	
Cryptogenic	61 (14.6)	19 (17.0)	42 (15.4)		15 (29.4)	46 (13.8)	
PSC	54 (12.9)	17 (15.2)	37 (13.6)		7 (13.7)	47 (14.1)	
Wilson disease	34 (8.1)	10 (8.9)	24 (8.8)		2 (3.9)	32 (9.6)	
HCV	14 (3.3)	5 (4.5)	9 (3.3)		2 (3.9)	12 (3.6)	
NASH	27 (6.4)	6 (5.4)	21 (7.7)		0 (0.0)	27 (8.1)	
Budd-Chiari syndrome	14 (3.3)	1 (0.9)	13 (4.8)		0 (0.0)	14 (4.2)	
Combination of several causes	29 (6.9)	9 (8.0)	20 (7.4)		5 (9.8)	24 (7.2)	
Having diabetes, n (%)	35 (8.4)	12 (9.4)	23 (7.9)	0.593^a^	4 (7.5)	31 (8.5)	> 0.999^b^
Having HBV, n (%)	106 (25.3)	43 (33.9)	63 (21.6)	**0.008** ^a^	12 (22.6)	94 (25.7)	0.634^a^
Having HCV, n (%)	37 (8.8)	14 (11.0)	23 (7.9)	0.297^a^	3 (5.7)	34 (9.3)	0.603^b^
Smoking, n (%)	35 (8.4)	15 (11.8)	20 (6.8)	0.092^a^	2 (3.8)	33 (9.0)	0.288^b^
Alcohol intake, n (%)	25 (6.0)	10 (7.9)	15 (5.1)	0.277^a^	0 (0.0)	25 (6.8)	0.058^b^
Family history of cancer, n (%)	38 (9.1)	13 (10.2)	25 (8.6)	0.583^a^	2 (3.8)	36 (9.8)	0.202^b^
Drugs after transplant, n (%)							
Cyclosporine	120 (28.6)	44 (34.6)	76 (26.0)	0.073^a^	18 (34.0)	102 (27.9)	0.359^a^
Tacrolimus	366 (87.4)	98 (77.2)	268 (91.8)	**<0.001** ^a^	40 (75.5)	326 (89.1)	**0.005** ^a^
Sirolimus	289 (69.0)	66 (52.0)	223 (76.4)	**<0.001** ^a^	31 (58.5)	258(70.5)	0.078^a^
Everolimus	57 (13.6)	15 (11.8)	42 (14.4)	0.480^a^	8 (15.1)	49 (13.4)	0.735^a^
Prednisolone	419 (100)						
Mycophenolate mofetil	410 (97.9)	126 (99.2)	284 (97.3)	0.288^b^	53 (100)	357 (97.5)	0.610^b^
Azathioprine	12 (2.9)	6 (4.7)	6 (2.1)	0.132^a^	6 (11.3)	6 (1.6)	**<0.001** ^a^
Age at transplantation (year), median (IQR)	39.00 (29.00-51.00)	44.00 (32.00-53.00)	37.50 (28.00-48.75)	**0.003** ^c^	43.00 (37.00-52.00)	38.00 (28.00-50.00)	**0.016** ^c^
BMI at transplantation (kg/m^2^), median (IQR)	23.38 (21.83-25.22)	23.44 (22.14-26.12)	23.36 (21.73-25.09)	0.303^c^	23.66 (21.86-24.52)	23.34 (21.82-25.39)	0.893^c^
MELD score at transplantation, median (IQR)	33.00 (29.00-37.00)	33.00 (29.00-37.00)	33.00 (28.00-36.00)	0.270^c^	31.00 (27.00-36.50)	33.00 (29.00-37.00)	0.147^c^

BMI: body mass index, HBV: hepatitis B virus, HCV: hepatitis C virus, IQR: interquartile range, MELD: model for end-stage liver disease, NASH: non-alcoholic steatohepatitis, PSC: primary sclerosing cholangitis. ^*^Comparison between alive and dead patients. ^†^Comparison between patients with and without *de novo* malignancy. Between groups analyses of data were conducted using ^(a)^ Chi-square, ^(b)^ Fisher’s exact test, or ^(c)^ Mann-Whitney U test. *P*-value < 0.05 was considered significant. There are some missing data. Bold *P* values denote significant differences. ^‡^Other causes of liver diseases: combination of several causes (n = 29), hepatocellular carcinoma (n = 10), primary biliary cholangitis (n = 6), acute liver failure (n = 4), biliary cirrhosis (n = 3), primary nonfunction (n = 2), neuroendocrine tumor (n = 2), alcohol intake (n = 2), cholangiocarcinoma (n = 1), rejection (n = 1), alpha-1 antitrypsin deficiency (n = 1), hemochromatosis (n = 1), hemangioma (n = 1), hyperoxaluria (n = 1).

 Among the included patients, 127 individuals (30.3%) died and 53 patients (12.6%) received a *de novo* solid malignancy diagnosis. Post-transplant lymphoproliferative disorders (37.73%), squamous cell carcinoma (13.21%), cholangiocarcinoma (9.43%), and basal cell carcinoma (9.43%) were the most prevalent diagnosed *de novo* solid malignancies diagnosed (Table 2).

**Table 2:** Frequency of Different Type of Cancers in Patients with *De Novo* Malignancy

**Type of malignancy**	**Frequency (%)**
Post-transplant lymphoproliferative disorders	20 (37.73)
Squamous cell carcinoma	7 (13.21)
Cholangiocarcinoma	5 (9.43)
Basal Cell Carcinoma	5 (9.43)
Lung cancer	3 (5.66)
Non-Hodgkin lymphoma	2 (3.77)
Colon cancer	2 (3.77)
Kaposi sarcoma	2 (3.77)
Germ cell tumor	2 (3.77)
Thyroid cancer	1 (1.87)
Prostate cancer	1 (1.87)
Breast cancer	1 (1.87)
Gastric adenocarcinoma	1 (1.87)

 When comparing the characteristics of alive and deceased patients, we observed that the patients who were alive had a lower age (*P *value = 0.003) and a lower prevalence of hepatitis B virus (21.6% *vs.* 33.9%, *P *value = 0.008), but a higher intake of tacrolimus (91.8% *vs.* 77.2%, *P *value < 0.001) and sirolimus (76.4% *vs.* 52%, *P *value < 0.001) compared to deceased patients. Furthermore, in the comparison between patients with and without* de novo* solid malignancies, significant differences were found in terms of etiology of liver disease (*P *value = 0.015), age (*P*-value = 0.016), as well as the use of tacrolimus (*P* value = 0.005) and azathioprine *(P *value < 0.001) (Table 1).

**Figure 2:**
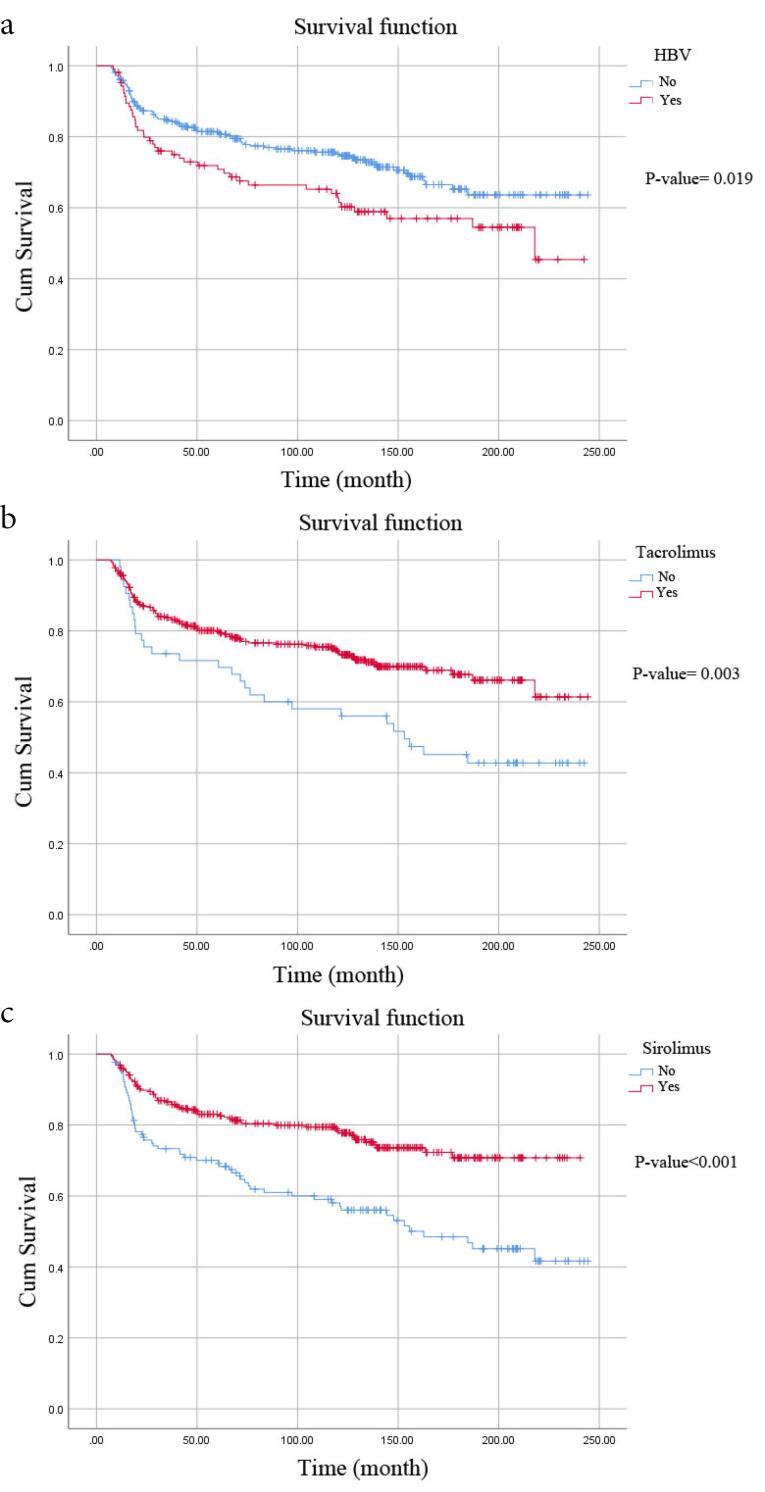
Comparison of Survival Rate According to Hepatitis B Virus Status (A), Tacrolimus Intake (B), and Sirolimus Intake (C)

**Figure 3:**
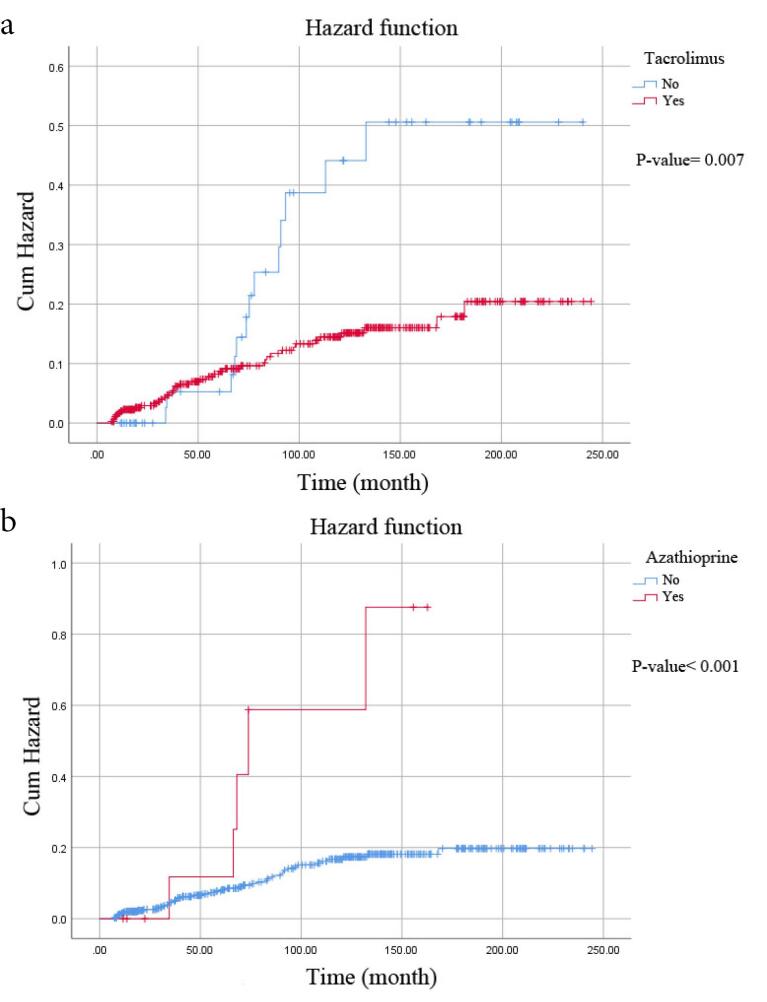
Comparison of time to de novo malignancy according to the Tacrolimus intake (A) and Azathioprine intake (B)

 The mean survival time was 175.72 months (95% CI: 165.93-185.52) for survival rate and 210.06 months (95% CI: 201.61-218.52) for *de novo* malignancy, according to the Kaplan-Meier method (the median survival period was 116.50 months). The life-table analysis demonstrated that the 1-, 5-, 10-, 15-, and 20-year survival rates of patients were 85%, 76%, 69%, 61%, and 58%, respectively. Furthermore, the 1-, 5-, 10-, 15-, and 20-year proportion of patients free from *de novo* malignancy were 97%, 90%, 83%, 78%, and 78%. According to the Log-Rank test, the survival rate was significantly lower among patients with hepatitis B (*P* value = 0.019) and those not using tacrolimus (*P* value = 0.003) or sirolimus (*P* value < 0.001) than their respective counterparts (Figure 2). Additionally, there were notable differences in the proportion of patients free from *de novo* malignancy between those who used tacrolimus (*P* value = 0.007) or azathioprine (*P* value < 0.001) compared to patients who did not use these medications (Figure 3).

 The Cox regression model indicated that age at transplantation was associated with poorer survival (HR = 1.03, 95% CI: 1.02 to 1.05, *P* value < 0.001), whereas sirolimus intake was identified as a significant predictor of better survival (HR = 0.44, 95% CI: 0.31-0.63, *P* value < 0.001). Furthermore, age (HR = 1.05, 95% CI: 1.02-1.07, *P* value < 0.001) and azathioprine (HR = 5.85, 95% CI: 2.47-13.87, *P* value < 0.001) were significantly linked to an increased risk of *de novo* solid malignancy (Table 3).

**Table 3:** Multivariable Cox Regression Model for Predictors of All-Causes Mortality and *De Novo *Malignancy Following Liver Transplantation

**Variables**	**HR (95% CI)**	***P *** **value**
Death^*^		
Age at transplantation (continuous)	1.03 (1.01 to 1.04)	< 0.001
Sirolimus (yes *vs.* no)	0.44 (0.31-0.63)	< 0.001
*De novo* malignancy^†^		
Age at transplantation (continuous)	1.05 (1.02 to 1.07)	< 0.001
Azathioprine (yes *vs.* no)	5.85 (2.47 to 13.87)	< 0.001

HR: hazard ratio, CI: Confidence Interval. ^*^Age, sex, smoking, body mass index, sirolimus, tacrolimus intake, and hepatitis B virus were included in the backward Cox. ^†^Age, sex, body mass index, and sirolimus, azathioprine, and tacrolimus intake were included in the backward Cox regression. Only the above variables remained in the final model. *P-value* < 0.05 was considered significant.

 Interaction tests for age and medication use showed no significant interactions (*P* > 0.05), indicating consistent effects across age groups. Subgroup analyses by sex and transplant indication also showed no significant differences.

## Discussion

 During our study period, 30.3% of the included patients died. The survival rate in the current analysis was almost similar to that reported in a previous meta-analysis study in Asia, which indicated one, two, three, five, and ten-year survival rates following liver transplantation at 85%, 80%, 75%, 73%, and 71%, respectively.^13^ Additionally, studies conducted in other regions reported similar estimations.^14^ Furthermore, we identified that patient age and sirolimus intake were predictors of patient survival following liver transplant in the multivariable model. Specifically, sirolimus use was associated with a 56% reduction in the mortality risk. Our findings are consistent with a recent meta-analysis indicating older recipients to have an increased mortality risk following liver transplant due to non-alcoholic steatohepatitis.^15^ Concerning sirolimus, a mammalian target of rapamycin inhibitor, Yan et al reported prolonged overall survival following sirolimus- or everolimus-based immunosuppression after liver transplantation for hepatocellular carcinoma in their pooled analyses of randomized controlled trials and cohort studies.^16^ The observed effects of sirolimus could be due to its beneficial effects on renal function, post-transplant hypertension, and antitumor activity.^17,18^ However, sirolimus is reported to have null or even detrimental effects on the survival rate in patients after liver transplantation for hepatitis.^19,20^ Contrary to our findings, some studies have shown that body mass index, diabetes, and the Model for End-Stage Liver Disease (MELD) score are potential risk factors limiting survival after liver transplantation.^15^ Discrepancies across studies may be partly attributed to differences in study designs, comorbidities, and underlying hepatic diseases. Further studies should be conducted in different health states with consideration of sirolimus’s adverse effects.

 One of the key findings in our study was that 12.6% of the included patients developed a* de novo* solid malignancy during the study period. The risk of *de novo* malignancy at 1-, 5-, 10-, and 20-year after liver transplantation was estimated to be 3%, 10%, 17%, and 22%, respectively. These figures were lower compared to a previous retrospective study based on the French national Agence de la Biomédecine database, which reported that 13.45% of adult liver transplant recipients developed *de novo* malignancies over a median follow-up period of approximately four years. Additionally, it was noted that the likelihood of *de novo* malignancy development after liver transplantation was 2.07% at 1 year, 13.30% at 5 years, and 28.01% at 10 years.^9^ In a separate study involving 174 Dutch adults, the cumulative risk for the development of *de novo* malignancy after liver transplantation was reported as 6%, 20%, and 55% at 5, 10 and 15 years post-transplantation, respectively.^21^ The variations in the rate of *de novo* malignancy following liver transplantation may be attributed to differences in study populations, geographical factors, underlying hepatic and associated conditions, duration of post-transplant follow-up, types of malignancies documented, methods used to identify malignancies, and the definition of* de novo* malignancy.

 In the present study, older patients were found to have a higher risk of developing *de novo* solid malignancy. Age was identified as an independent predictor of *de novo* malignancy following liver transplantation in some previous studies^9,22^ but not all.^23^ Furthermore, azathioprine use was associated with a substantially increased risk of *de novo* malignancy following liver transplantation, representing a nearly 6-fold higher incidence compared to non-exposed patients. Consistent with our findings, a prior meta-analysis demonstrated that patients receiving azathioprine after solid organ transplantation had significantly greater odds of developing *de novo* carcinoma compared to those on mycophenolate.^6^ Various mechanisms have been proposed to explain these observations. The carcinogenic effects of azathioprine may be attributed to its immunosuppressive actions on T cells and macrophages, its potential to induce mutations in human cells, and its interference with DNA repair mechanisms.^24^ Further studies are warranted to elucidate the underlying mechanisms more comprehensively.

 Our center is one of the largest liver transplant centers in the world, providing a unique opportunity to evaluate long-term outcomes with good generalizability. However, our study had several limitations, including the retrospective design, single-center design with low generalizability, lack of time-updated exposure modeling, absence of cause-specific mortality data, small number of events that limits subgroup analyses, and inclusion of patients with various types of liver disease. Furthermore, the exact dose and duration of medications were inconsistently documented and thus not analyzed.

 In conclusion, 12.6% of the included patients developed a *de novo* malignancy during the study period. Post-transplant lymphoproliferative disorders were found to be the most prevalent malignancy. Age and immunosuppressive regimen were independently associated with mortality and malignancy development following liver transplantation. Our findings highlight the need for further studies to evaluate potential benefits of screening strategies in high-risk recipients.
